# Draft Genome Sequence of *Pediococcus pentosaceus* Strain FBL2, a Probiotic Bacterium Isolated from Jogaejeot, a Salted Fermented Food, in the Republic of Korea

**DOI:** 10.1128/genomeA.00303-17

**Published:** 2017-05-04

**Authors:** Eiseul Kim, Jae-Hwan Kim, Saet-Byul Park, Mi-ju Kim, Hyun-Joong Kim, Chang-Gyeom Kim, Dong-Won Choo, Hae-Yeong Kim

**Affiliations:** a Institute of Life Sciences and Resources Graduate School of Biotechnology, Kyung Hee University, Yongin, Republic of Korea; b Department of Bioinformatics and Biosystems, Korea Polytechnics, Seongnam, Republic of Korea

## Abstract

*Pediococcus pentosaceus* strain FBL2 is a lactic acid bacterium isolated in the Republic of Korea from jogaejeot, a salted fermented food made with shellfish. *P. pentosaceus* strain FBL2 comprised 54 contigs (≥1 kb) and had a total draft genome size of 1,934,229 bp with a G+C content of 37.2%.

## GENOME ANNOUNCEMENT

*Pediococcus pentosaceus* is a Gram-positive, coccus-shaped, nonmotile, and non-spore-forming lactic acid bacterium (LAB). *P. pentosaceus* strains are frequently isolated from bacterial ripened cheese, plant materials, and dairy and meat products, and they are used industrially as starter cultures for fermented foods (1–3). The genomes of two *P. pentosaceus* strains—ATCC 25745 (CP000422.1) and SL4 (CP006854.1)—were previously sequenced and annotated (4, 5). The complete genome of *P. pentosaceus* strain ATCC 25745 consisted of 1,832,387 nucleotides and 1,747 protein-coding genes, and the complete genome of *P. pentosaceus* strain SL4 consisted of 1,789,138 nucleotides and 1,709 protein-coding genes.

*P. pentosaceus* strain FBL2 was isolated in the Republic of Korea from commercial jogaejeot, a common salted fermented food made with shellfish, and the 16S rRNA gene sequence of the strain showed 100% identity with that of *P. pentosaceus* strain wg2 (KC352727.1).

Genomic DNA was extracted with the G-spin genomic extraction kit (Intron Biotechnology Co., Republic of Korea) and its quality was evaluated using an Agilent 2100 bioanalyzer with the high-sensitivity DNA kit. Draft genome sequencing was performed using an Ion Torrent Personal Genome Machine (Life Technologies, Inc., Germany) and a 318 semiconductor chip with 400-bp sequencing reads. A total of 4,444,435 reads, with an average read length of 280 bp, were obtained. Reference-based assembly was carried out using SPAdes version 3.1.0 with *P. pentosaceus* ATCC 25745 (CP000422.1) as the reference genome. Assembly of the reads resulted in 54 contigs ≥1 kb (1 to 290,155 kb; *N*_50_ length 91,622 bp). The total draft genome size was 1,934,229 bp with a G+C content of 37.2%.

The genome sequence was annotated with Rapid Annotations using Subsystems Technology (RAST) (6, 7). The draft genome had 1,898 coding sequences, 55 RNAs, and 289 subsystems. Genes involved in bile tolerance (choloylglycine hydrolase), acid tolerance (F1F0-ATPase system), oxidative stress resistance (NADH-peroxidase, similar to glutathione reductase), colicin V synthesis, and antibiotic resistance (fluoroquinolone and beta-lactamase) were present in the genome.

The following were also detected in the *P. pentosaceus* strain FBL2 genome: ATP-binding protein OpuCA, permease-binding proteins OpuCB and OpuCD, substrate-binding protein OpuCC, osmotically activated l-carnitine/choline ABC transporter, fibronectin-binding protein (which plays an essential role in bacterial adhesion), asparagine synthetase, and beta-lactamase class A gene. Compared to the *P. pentosaceus* ATCC 25745 genome, approximately 58 genes were missing.

### Accession number(s).

This whole-genome shotgun project has been deposited in DDBJ/ENA/GenBank under the accession number LSFE00000000. The version described in this paper is the first version, LSFE01000000.
